# Prognostic significance of PD-L1 expression and CD8+ T cell infiltration in pulmonary neuroendocrine tumors

**DOI:** 10.1186/s13000-018-0712-1

**Published:** 2018-05-22

**Authors:** Haiyue Wang, Zhongwu Li, Bin Dong, Wei Sun, Xin Yang, Ruping Liu, Lixin Zhou, Xiaozheng Huang, Ling Jia, Dongmei Lin

**Affiliations:** 1 Key Laboratory of Carcinogenesis and Translational Research (Ministry of Education), Department of Pathology, Peking University Cancer Hospital & Institute, No. 52 Fucheng Road, Haidian District, Beijing, 100142 People’s Republic of China; 2 Key Laboratory of Carcinogenesis and Translational Research (Ministry of Education), Department of Central Laboratory, Peking University Cancer Hospital & Institute, Beijing, 100142 People’s Republic of China; 3 Beijing Institute of Graphic Communication, Beijing, 102600 People’s Republic of China

**Keywords:** Pulmonary neuroendocrine tumor, PD-L1, CD8+ TILs

## Abstract

**Background:**

Recent research supports a significant role of immune checkpoint inhibitors in the treatment of solid tumors. However, relevant reports for programmed death-ligand 1 (PD-L1) and CD8+ tumor-infiltrating lymphocytes (TILs) in pulmonary neuroendocrine tumors (PNETs) have not been fully studied. Therefore, we investigated PNETs for the expression of PD-L1 and infiltration by CD8+ TILs as well as the prognostic value of both factors.

**Methods:**

In total, 159 specimens of PNETs (35 TC, 2 AC, 28 LCNEC, 94 SCLC) were included in this study. Immunohistochemistry (IHC) was used to detect the expression of PD-L1 in these cases. Cases demonstrating ≥5% tumor cell expression or any expression (> 1%) of PD-L1 on immune cells were considered positive. CD8+ TILs both within stroma and tumor areas of invasive carcinoma were analyzed using whole-slide digital imaging. Manual regional annotation and machine cell counts were performed for each case.

**Results:**

Positive expression of PD-L1 was observed in 72 cases (45.3%), including 9 cases (5.7%) with expression exclusively on tumor cells, 46 cases (28.9%) with expression exclusively on immune cells, and 17 cases (10.7%) with the expression on tumor cells and immune cells. PD-L1 expression was associated with necrosis (*p* < 0.001), high pathologic grade (*p* < 0.001) and histologic type (*p* < 0.001). No correlation was observed with overall survival (OS) (*p* = 0.158) or progression-free survival (PFS) (*p* = 0.315). In contrast, higher CD8+ T cell density was associated with the absence of vascular invasion (*p* = 0.004), histologic type (*p* = 0.005), negative lymph node metastasis (*p* = 0.005) and lower clinical staging (*p* = 0.007). Moreover, multivariate analysis revealed that CD8+ stromal TIL was an independent prognostic factor for improved OS (*p* = 0.009) and PFS (*p* = 0.002).

**Conclusion:**

PD-L1 was expressed in approximately half of the PNETs. The majority of the expression was observed in immune cells. Positive expression of PD-L1 showed no correlation with OS or PFS, while higher CD8+ TILs within stroma was proved to be an independent prognostic factor for favorable OS and PFS of PNETs.

## Background

Neuroendocrine lung tumors represent a spectrum of low-grade typical carcinoids (TC), atypical carcinoids (AC), high-grade large cell neuroendocrine carcinoma (LCNEC) and small cell lung carcinoma (SCLC). SCLC is the most aggressive and most common of all malignant neuroendocrine tumors, with an incidence of 15–20%. LCNEC represents approximately 3% of lung tumors. TC accounts for 1–2%, while AC is the rarest of the lung neuroendocrine tumors (0.1–0.2%) [1–3]. Traditional treatments for high-grade malignancy, which include chemotherapy and radiation, have remained unchanged for decades. Prognosis remains dismal for these patients [4]. Therefore, to improve the prognosis of PNETs, a promising therapeutic strategy is urgently needed.

In the process of tumorigenesis, neoplastic transformation alters the structure of the normal tissue, leading to the activation of effector T cells, which then seeks to eliminate the transformed cells. Novel immunotherapy targeting immune checkpoint molecules such as programmed death-ligand 1 (PD-L1), based on the mechanism of cancer-immune escape, have successfully garnered increasing attention. Programmed death ligand 1 (PD-L1; also called B7-H1 or CD274) can be detected in many cancer cells and immune cells including the antigen-presenting cells (APCs) [5]. During the development of human cancer, the PD-L1 molecule is abnormally activated and overexpressed, which may suppress T cell migration, proliferation, secretion of cytotoxic mediators, and restrict cancer cell killing [6]. Therefore, monoclonal antibodies (mAbs) blocking the PD-L1 pathway or immunomodulatory agents with similar effects may improve anticancer immunity via enhancing T cell functions. Monoclonal antibodies targeting PD-L1 or PD-1 are currently being investigated in clinical trials. The results obtained to date have demonstrated remarkable clinical responses in many different types of cancer [6–10].

Cytotoxic lymphocytes (CTLs), which play a critical role in the anticancer response, are actively suppressed in the tumor microenvironment [11]. Some CTLs manage to be released into circulation, then migrate into tumor tissue through complicated interactions with adhesion receptors, at which point they are called tumor-infiltrating lymphocytes (TILs) [12]. Given that PD-L1 on tumor cells or immune cells interacts with CD8 expressed on T cells in the tumor environment, a comprehensive analysis of CD8/PD-L1 related molecules might provide important information for determining the clinical relevance to the neuroendocrine tumors of the lung. However, to the best of our knowledge, expression of PD-L1 on PNETs has not been fully studied [13].

In this study, we investigated the pattern of PD-L1 expression and the density of CD8+ TILs in PNETs. Moreover, we analyzed the clinicopathological characteristics with the expression of PD-L1 and the number of CD8+ TIL levels. We also performed a survival analysis to determine the correlation of PD-L1 expression and CD8+ TIL counts with OS and PFS.

## Methods

### Patient cohort

A total of 159 patients with PNETs who underwent pulmonary lobectomy or lymph node resection at Peking University Cancer Hospital (Beijing, China) from 2010 to 2015 were included in this study. Of those cases, 35 were TC, 2 were AC, 28 were LCNEC, and 94 were SCLC. All cases were reviewed by two experienced pathologists (Zhongwu Li and Bin Dong) to confirm the diagnosis based on the current WHO criteria for neuroendocrine lung tumors. The staging was undertaken according to the 8th edition AJCC tumor, lymph node, metastasis (TNM) classification. All the clinical characteristics (including age, gender, histologic type, necrosis, vascular invasion, preoperative chemotherapy, lymph node metastasis, and clinical TNM staging) were collected.

### Immunohistochemistry

Serial sections with a thickness of 4 μm from the whole formalin-fixed paraffin-embedded (FFPE) samples of PNETs were cut onto glass slides, followed by IHC staining. Evaluation of PD-L1 was performed using a rabbit anti-PD-L1 monoclonal antibody (clone SP142; ZSGB-BIO, Beijing, China) at a working solution and incubated for 15 min at 37 °C on an autostainer (BOND-MAX, LEICA, Leica Biosystems Newcastle Ltd., Newcastle, UK). Cases demonstrating ≥5% tumor cell expression (membrane) or any expression (>1%) of PD-L1 on immune cells (membrane or cytoplasm) were considered positive [14]. Slides stained with CD8 were labeled by a rabbit anti-CD8 monoclonal antibody (clone SP16; ZSGB-BIO, Beijing, China) at a working solution and incubated for 36 min at 37 °C on an autostainer (BenchMark ULTRA, Roche, Ventana Medical Systems, Oro Valley, AZ, USA). For each case, manual regional annotation and machine cell counts were used to measure cell density in intratumoral as well as stromal areas of invasive carcinoma. CD8+ TIL density was analyzed by whole slide digital scanning using an Digital Pathology Scanner (Aperio VERSA, Leica Biosystems, Buffalo Grove, IL, USA), and the scoring was assessed on an Aperio Scanscope (Aperio Technologies; USA) by the method of rare event tissue test. Positive cell counts were measured in 4 peritumoral and 6 intratumoral non-overlapping fields using fixed areas of 1.44 square millimeters. The grading system was defined as two groups according to the median CD8+ T cell density in stroma and intratumor.

### Statistical analysis

SPSS 17.0 version (IBM Corporation, Armonk, NY, USA) was used for all statistical analyses. Correlations between CD8+ T cell density and PD-L1 expression with the clinicopathological variables were performed using the Pearson’s chi-square and Fisher’s exact test. Survival analysis was performed using the univariate Kaplan-Meier method. Multivariate analysis was performed with the Cox proportional hazards model. Overall survival was calculated from the date of pathological diagnosis to time of death or last follow-up. Progression-free survival was calculated from date of pathological diagnosis to time of last clinical evidence of recurrence, progression, or death. Two-sided *p* value < 0.05 was considered statistically significant.

## Results

### Patient clinicopathological characteristics and PD-L1 immunoreactivity

The clinicopathological features of 159 patients diagnosed as neuroendocrine tumors of lung are presented in Table 1. The study included 109 males (68.6%) and 50 females (31.4%). Median age was 59.5 years (range, 30 to 83). A small minority of patients (5.7%) had received neoadjuvant chemotherapy before surgery, while 148 cases did not. For most cases, the primary site was in the lung (*n* = 131, 82.4%). Of the 159 samples, most tumors showed no vascular invasion (*n* = 129, 81.1%). Approximately half of the cases had tumor necrosis (*n* = 74, 46.5%). Lymph node metastasis was detected in 63 patients (47.8%). Tumors were classified as stage I (*n* = 70, 48.6%), stage II (*n* = 27, 18.8%), or stage III (*n* = 47, 32.6%).

**Table 1:** Clinical characteristics in the four subtypes

Variable	All tumors	TC	AC	LCNEC	SCLC	NA
	159 (100.0)	35 (100.0)	2 (100.0)	28 (100.0)	94 (100.0)	
Age						
< =59.5	78 (49.1)	25 (71.4)	2 (100.0)	12 (42.9)	39 (41.5)	
> 59.5	81 (50.9)	10 (28.6)	0 (0.0)	16 (57.1)	55 (58.5)	
Gender						
Male	109 (68.6)	17 (48.6)	0 (0.0)	23 (82.1)	69 (73.4)	
Female	50 (31.4)	18 (51.4)	2 (100.0)	5 (17.9)	25 (27.6)	
Necrosis						
No	85 (53.5)	35 (100.0)	0 (0.0)	9 (32.1)	41 (43.6)	
Yes	74 (46.5)	0 (0.0)	2 (100.0)	19 (67.9)	53 (56.4)	
Vascular invasion						
No	129 (81.1)	34 (97.1)	1 (50.0)	22 (78.6)	72 (76.6)	
Yes	30 (18.9)	1 (2.9)	1 (50.0)	6 (21.4)	22 (23.4)	
Preoperative therapy						
No	150 (94.3)	35 (100.0)	2 (100.0)	27 (96.4)	86 (91.5)	
Yes	9 (5.7)	0 (0.0)	0 (0.0)	1 (3.6)	8 (8.5)	
First location						
Lung	131 (82.4)	35 (100.0)	2 (100.0)	28 (100.0)	66 (70.2)	
Lymph Node	28 (17.6)	0 (0.0)	0 (0.0)	0 (0.0)	28 (29.8)	
Lymph Node Metastasis						13 (8.2)
No	83 (52.2)	32 (97.0)	1 (50.0)	14 (60.9)	36 (40.9)	
Yes	63 (47.8)	1 (3.0)	1 (50.0)	9 (39.1)	52 (59.1)	
Clinical Staging						15 (9.4)
I	70 (48.6)	30 (90.9)	1 (50.0)	10 (43.5)	29 (33.7)	
II	27 (18.8)	2 (6.1)	0 (0.0)	9 (39.1)	16 (18.6)	
III	47 (32.6)	1 (3.0)	1 (50.0)	4 (17.4)	41 (47.7)	
Patterns of PD-L1						
Tumor^pos^ stroma^neg^	9 (5.7)	3 (8.6)	0 (0.0)	5 (17.8)	1 (1.1)	
Stroma^pos^tumor^neg^	46 (28.9)	0 (0.0)	0 (0.0)	12 (42.9)	34 (36.2)	
Tumor and stroma^pos^	17 (10.7)	0 (0.0)	0 (0.0)	4 (14.3)	13 (13.8)	
Tumor and stroma^neg^	87 (54.7)	32 (91.4)	2 (100.0)	7 (25.0)	46 (48.9)	
CD8 density/mm^2^ in stroma						
≤ 264.6/mm^2^	80 (50.3)	23 (65.7)	0 (0.0)	7 (25.0)	50 (53.2)	
> 264.6/mm^2^	79 (49.7)	12 (34.3)	2 (100.0)	21 (75.0)	44 (46.8)	

*Abbreviation*: *NA* not available

PD-L1 was positively expressed in 72 cases (45.3%), including 9 with expression exclusively on tumor cells (5.7%), 46 with expression exclusively on immune stromal cells (28.9%), and 17 with expression both on tumor cells and immune cells (10.7%). Membranous and cytoplasmic expression of PD-L1 protein was detected in four types of PNETs, including SCLC (*n* = 48, 66.7%), LCNEC (*n* = 21, 29.2%), AC (*n* = 0, 0.0%), and TC (*n* = 3, 4.1%).

### Association between PD-L1 expression and clinicopathologic parameters

Associations between PD-L1 and clinical parameters in patients with PNETs are described in Table 2. The expression of PD-L1 was significantly associated with the presence of tumor necrosis (*p* < 0.001), high pathologic grade (*p* < 0.001), and histologic type (particularly for LCNEC) (*p* < 0.001). There was no significant association between PD-L1 and age (*p* = 0.241), gender (*p* = 0.365), prior neoadjuvant therapy (*p* = 0.538), vascular invasion (*p* = 0.072), lymph node metastasis (*p* = 0.170), or clinical staging (*p* = 0.314). The pattern of PD-L1 expression was reclassified as tumor-positive and stroma-positive. Statistical correlations between tumor cell or immune cell and clinical parameters are present in Table 3. The PD-L1 positive expression in immune cells was associated with tumor necrosis (*p* < 0.001), high pathologic grade (*p* < 0.001), and histologic type (particularly for LCNEC) (*p* < 0.001).

**Table 2:** The association between PD-L1 expression, CD8+ T cell infiltration and clinicopathologic parameters in PNETs

Variable		PD-L1 expression			CD8+ T cell density in stroma			NA
	N(%)	Positive	Negative	*p*	≤264.6/mm^2^	>264.6/mm^2^	*p*	
Total	159 (100.0)	72 (45.3)	87 (54.7)		80 (50.3)	79 (49.7)		
Age				0.241			0.578	
≤ 59.5	78 (49.1)	39 (54.2)	39 (44.8)		41 (51.3)	37 (46.8)		
>59.5	81 (50.9)	33 (45.8)	48 (55.2)		39 (48.7)	42 (53.2)		
Gender				0.365			0.189	
Male	109 (68.6)	52 (72.2)	57 (65.5)		51 (63.8)	58 (73.4)		
Female	50 (31.4)	20 (27.8)	30 (34.5)		29 (36.3)	21 (26.6)		
Necrosis				< 0.001*			0.096	
No	85 (53.5)	17 (23.6)	68 (78.2)		48 (60.0)	37 (46.8)		
Yes	74 (46.5)	55 (76.4)	19 (21.8)		32 (40.0)	42 (53.2)		
Vascular invasion				0.072			0.004*	
No	129 (81.1)	54 (75.0)	75 (86.2)		72 (90.0)	57 (72.2)		
Yes	30 (18.9)	18 (25.0)	12 (13.8)		8 (10.0)	22 (27.8)		
Preoperative therapy				0.538			0.113	
No	148 (93.1)	68 (94.4)	80 (92.0)		77 (96.3)	71 (89.8)		
Yes	11 (6.9)	4 (5.6)	7 (8.0)		3(3.7)	8 (10.2)		
Tumor type				< 0.001*			0.005*	
SCLC	94 (59.1)	48 (51.1)	46 (48.9)		50 (53.2)	44 (46.8)		
LCNEC	28 (17.6)	21 (75.0)	7 (25.0)		7 (25.0)	21 (75.0)		
TC	35 (22.0)	3 (8.6)	32 (91.4)		23 (65.7)	12 (34.3)		
AC	2 (1.3)	0 (0.0)	2 (100.0)		0 (0.0)	2 (100.0)		
Pathological grading				< 0.001*			0.100	
High	122 (76.7)	69 (95.8)	53 (60.9)		57 (71.2)	65 (82.3)		
Low	37 (23.3)	3 (4.2)	34 (39.1)		23 (28.8)	14 (17.7)		
Lymph node metastasis				0.170			0.005*	13 (8.2)
No	83 (56.8)	34 (50.7)	49 (62.8)		33 (45.2)	50 (68.5)		
Yes	63 (43.2)	33 (49.3)	30 (37.2)		40 (54.8)	23 (31.5)		
Clinical Staging				0.314			0.007*	15 (9.4)
I	70 (48.6)	29 (41.4)	41 (58.6)		27 (38.6)	43 (61.4)		
II	27 (18.8)	10 (37.0)	17 (63.0)		14 (51.9)	13 (48.1)		
III	47 (32.6)	25 (53.2)	22 (46.8)		32 68.1)	15 (31.9)		

*Abbreviation*: *NA* not available

*stands for the value of *p* < 0.05

**Table 3:** The expression patterns of PD-L1 to clinicopathologic parameters in PNETs

		PD-L1 expression						NA
	N(%)	Tumor^pos^	Tumor^neg^	*p*	Stroma^pos^	Stroma^neg^	*p*	
Total	159 (100.0)	26 (16.4)	133 (83.6)		63 (39.6)	96 (60.4)		
Age				0.593			0.497	
≤ 59.5	78 (49.1)	14 (53.8)	64 (48.1)		33 (52.4)	45 (46.9)		
>59.5	81 (50.9)	12 (46.2)	69 (51.9)		30 (47.6)	51 (53.1)		
Gender				0.142			0.527	
Male	109 (68.6)	21 (80.8)	88 (66.2)		45 (71.4)	64 (66.7)		
Female	50 (31.4)	5 (19.2)	45 (33.8)		18 (28.6)	32 (33.3)		
Necrosis				0.094			< 0.001*	
No	85 (53.5)	10 (38.5)	75 (56.4)		11 (17.5)	74 (77.1)		
Yes	74 (46.5)	16 (61.5)	58 (43.6)		52 (82.5)	22 (22.9)		
Vascular invasion				0.251			0.088	
No	129 (81.1)	19 (73.1)	110 (82.7)		47 (74.6)	82 (85.4)		
Yes	30 (18.9)	7 (26.9)	23 (17.3)		16 (25.4)	14 (14.6)		
Preoperative therapy				0.865			0.819	
No	148 (93.1)	24 (92.3)	124 (93.2)		59 (93.7)	89 (92.7)		
Yes	11 (6.9)	2 (7.7)	9 (6.8)		4 (6.3)	7 (7.3)		
Tumor type				0.066			< 0.001*	
SCLC	94 (59.1)	14 (14.9)	80 (85.1)		47 (50)	47 (50)		
LCNEC	28 (17.6)	9 (32.1)	19 (67.9)		16 (57.1)	12 (42.9)		
TC	35 (22.0)	3 (8.6)	32 (91.4)		0 (0.0)	35 (100.0)		
AC	2 (1.3)	0 (0.0)	2 (100.0)		0 (0.0)	2 (100.0)		
Pathological grading				0.122			< 0.001*	
High	122 (76.7)	23 (88.5)	99 (74.4)		63 (100.0)	59 (61.5)		
Low	37 (23.3)	3 (11.5)	34 (25.6)		0 (0.0)	37 (38.5)		
Lymph node metastasis				0.377			0.113	13 (8.2)
No	83 (52.2)	15 (65.2)	68 (55.3)		30 (49.2)	53 (62.4)		
Yes	63 (47.8)	8 (34.8)	55 (44.7)		31 (50.8)	32 (37.6)		
Clinical Staging				0.309			0.077	15 (9.4)
I	70 (48.6)	14 (63.6)	56 (45.9)		25 (43.1)	45 (52.3)		
II	27 (18.8)	3 (13.6)	24 (19.7)		8 (13.8)	19 (22.1)		
III	47 (32.6)	5 (22.7)	42 (34.4)		25 (43.1)	22 (25.6)		
CD8 density/mm^2^ in stroma				0.002*			0.005*	
≤ 264.6/mm^2^	80 (50.3)	6 (23.1)	74 (55.6)		23 (36.5)	57 (59.4)		
>264.6/mm^2^	79 (49.7)	20 (76.9)	59 (44.4)		40 (63.5)	39 (40.6)		

*Abbreviation*: *NA* not available

*stands for the value of *p* < 0.05

### Correlations among CD8+ T cell infiltration and clinicopathologic parameters

CD8+ TILs grading was subdivided into two groups based on the median CD8+ T cell density within stroma (264.6/mm^2^) in PNETs (Table 2). Higher CD8+ T cell density (> 264.6/mm^2^) was associated with the absence of vascular invasion (*p* = 0.004), histologic type (particularly for AC) (*p* = 0.005), negative lymph node metastasis (*p* = 0.005), and lower clinical staging (*p* = 0.007). No correlation was observed between CD8+ TILs and age, gender, necrosis or neoadjuvant therapy.

**Fig. 1:**
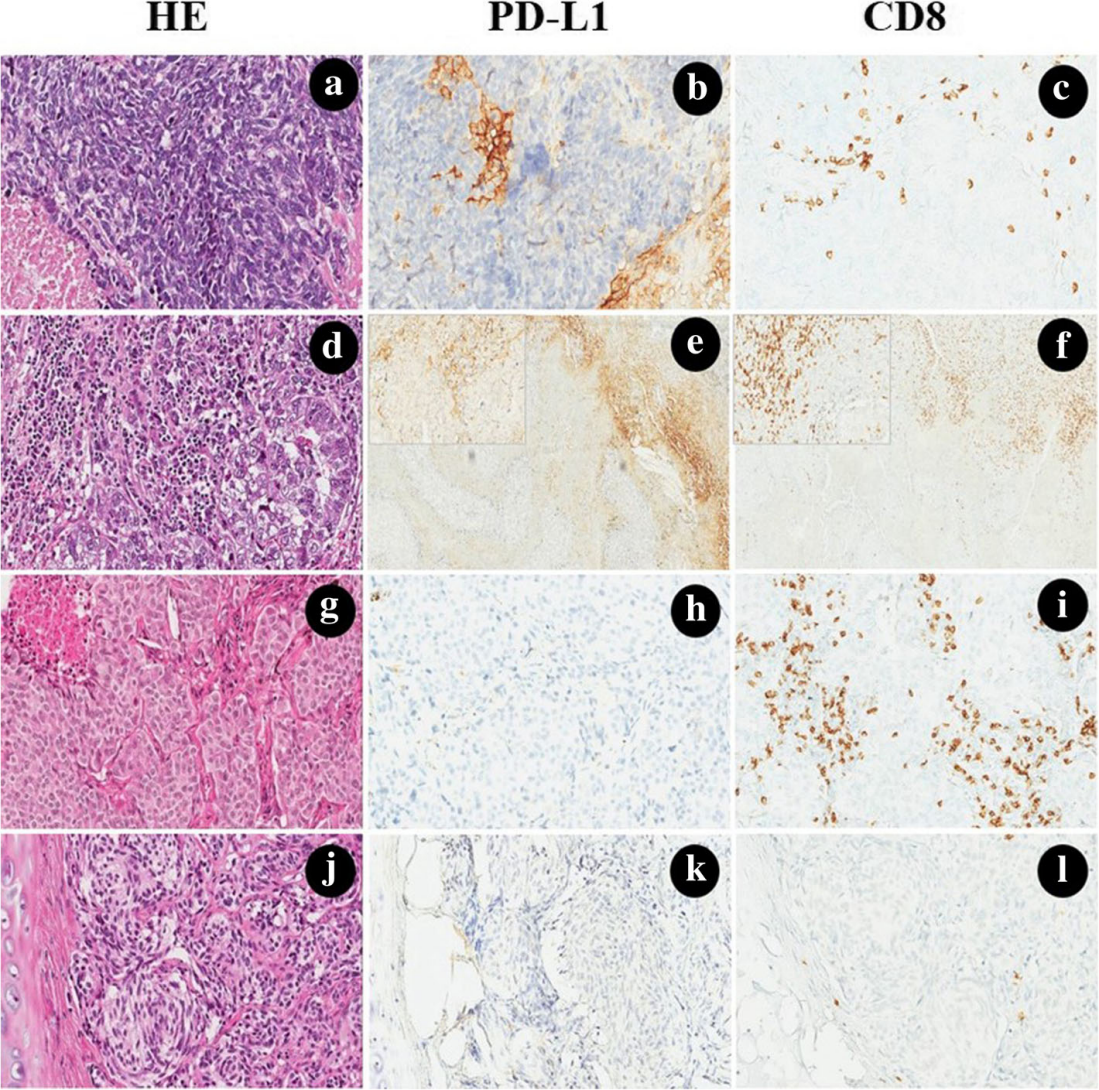
Hemotoxylin and eosin (H&E), PD-L1 and CD8 stains, each performed on histologic sections of small cell lung cancer (**a**-**c**), large cell neuroendocrine carcinoma (**d**-**f**), atypical carcinoid (**g**-**i**) and typical carcinoid (**j**-**l**). **a** Small cell lung cancer was showing a scant cytoplasm, fine nuclear chromatin, absent or inconspicuous nucleoli, extensive necrosis. **b** PD-L1 was moderately expressed on the membrane of stromal immune cells in the desmoplastic stroma between clusters of tumor cells. **c** CD8+ TILs were observed in the stroma, while the intratumoral pattern of CD8 expression was not common. **d** Large cell neuroendocrine carcinoma with prominent nucleoli and abundant eosinophilic cytoplasm, necrosis was not shown. **e**, **f** PD-L1 was positively expressed on the membrane and cytoplasm of the immune cells, and a large number of CD8+ TILs could also be observed at the borderline (the 200× magnification for PD-L1 and CD8 was shown in the upper left, respectively). **g** Atypical carcinoid with vascularized stroma, focal necrosis and 6 mitosis/2 mm^2^. **h**, **i** PD-L1 was negative expression either in tumor cells or stromal cells, while CD8+ TILs were exhibited in the interface of tumor and stroma. **j** Typical carcinoid with organoid growth pattern with intervening vascular stroma. **k**, **l** No PD-L1 can be detected, and only several CD8+ TILs could be found in the stroma. (The original magnification of **e-f** was 100×, magnification for remaining cases were 200×)

PD-L1 expression was positively correlated with CD8+ TIL expression within stroma. Figure 1 presents the patterns of PD-L1 expression and CD8+ T cell infiltration in high-grade and low-grade malignancies.

### Association of PD-L1 expression and CD8+ TILs with OS and PFS

**Fig. 2:**
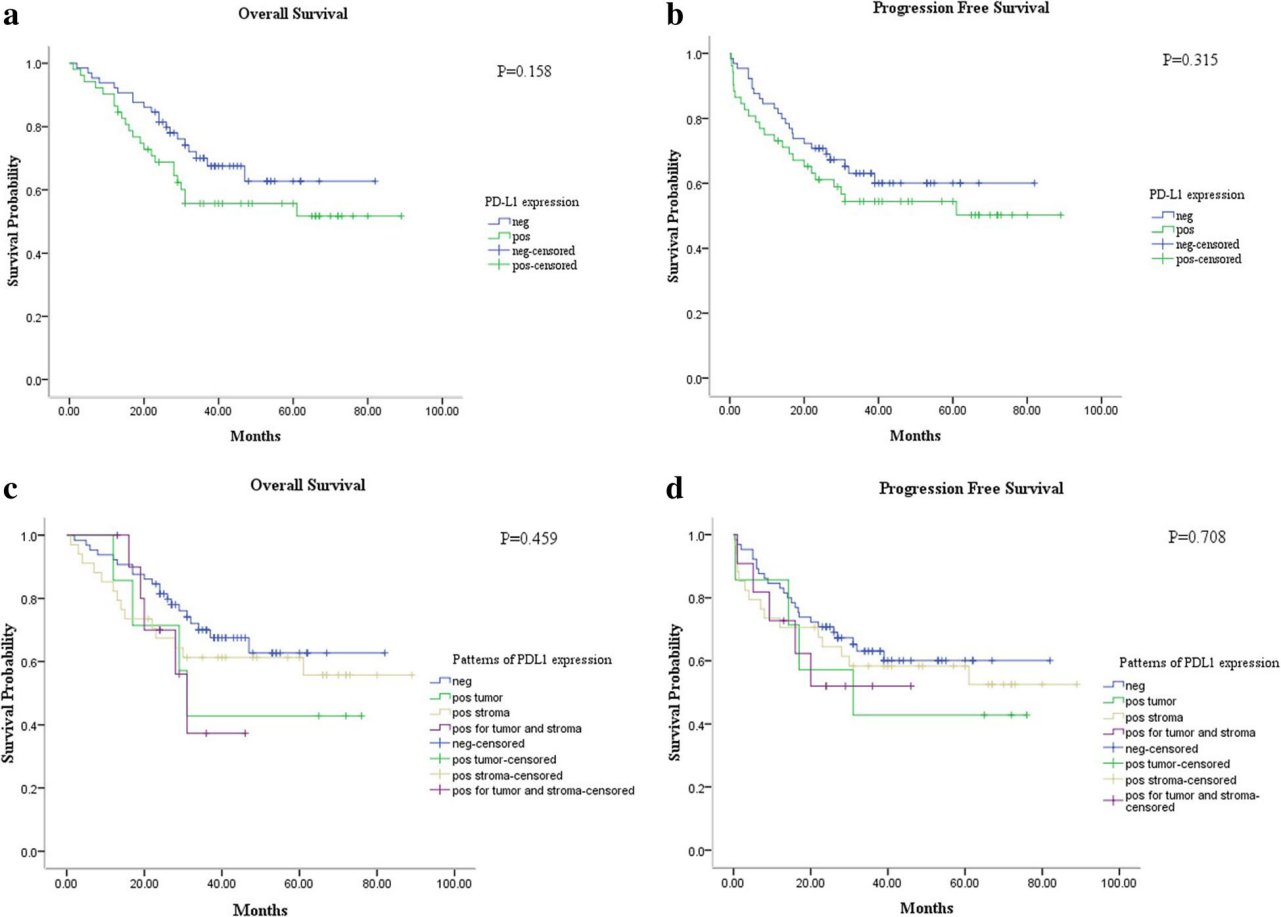
Kaplan-Meier survival curves of OS and PFS for PD-L1 were performed. The PD-L1 expression has a trend with decreased OS (*p* = 0.158) (**a**) and PFS (*p* = 0.315) (**b**) but did not reach the significant level. Similarly, the PD-L1 positive target cells were further classified as tumor cells and immune cells. We found that expression on tumor cells seemed to have poorer OS (*p* = 0.459) (**c**) and PFS (*p* = 0.708) (**d**) than in immune cells

Univariate survival analysis showed a trend toward an association between positive expression of PD-L1 in patients with PNETs and decreased OS (*p* = 0.158) and PFS (*p* = 0.315). However, this trend did not reach the level of statistical significance. Similarly, expression of PD-L1 on tumor cells showed a related trend toward poorer OS (*p* = 0.459) and PFS (*p* = 0.708) (Fig. 2).

**Fig. 3:**
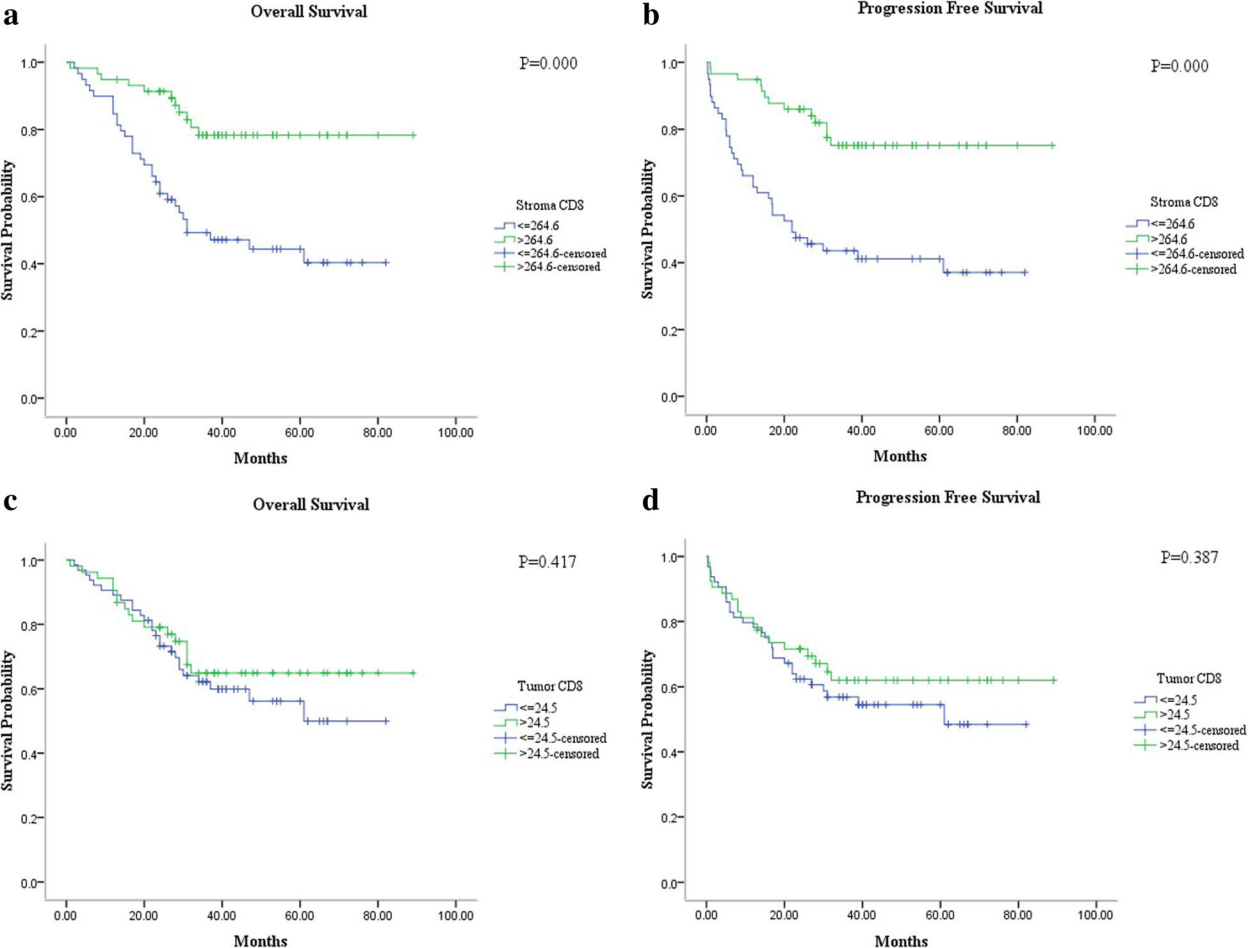
In PNETs, Kaplan-Meier analysis of OS and PFS associations with CD8+ T cell density within stroma (**a**-**b**) and intratumor (**c**-**d**) were performed. Increasing stromal CD8+ T cell density is correlated with improved OS (*p* = 0.000) (**a**) and PFS (*p* = 0.000) (**b**), while the higher intratumoral CD8+ T cell density was associated with a trend toward improved prognosis (**c** and **d**)

We also evaluated the relationship of CD8+ TIL density in stromal and intratumoral areas with OS and PFS. Immune stromal CD8+ T cell density was classified as low (≤264.6/mm^2^) or high (>264.6/mm^2^). Intratumoral CD8+ T cell density was also subdivided as low (≤24.5/mm^2^) or high (>24.5/mm^2^). The presence of high CD8+ TIL density in stroma proved to be a better prognostic factor for improved OS (*p* = 0.000) and PFS (*p* = 0.000). No correlation between intratumoral CD8+ T cell density and OS (*p* = 0.417) or PFS (*p* = 0.387) was observed (Fig. 3).

For multivariate analysis, the Cox proportional hazards model was performed and included age, gender, necrosis, vascular invasion, lymph node metastasis, clinical staging, PD-L1 expression and CD8+ TILs in the stroma. The results revealed that expression of PD-L1 was not an independent prognostic factor for OS (Hazard ratio [HR] = 0.707; 95% confidence interval [CI], 0.367–1.364; *p* = 0.301) or PFS (Hazard ratio [HR] = 0.921; 95% confidence interval [CI], 0.420–2.021; *p* = 0.838). In contrast, the presence of high CD8+ TIL density in stroma was an independent prognostic factor for improved OS (Hazard ratio [HR] = 2.770; 95% confidence interval [CI], 1.294–5.930; *p* = 0.009) and PFS (Hazard ratio [HR] = 3.011; 95% confidence interval [CI], 1.492–6.079; *p* = 0.002).

## Discussion

In this study, we investigated the associations between PD-L1 and other clinicopathologic characteristics. Our examination of PNETs showed that expression of PD-L1 was significantly associated with high pathologic grade, advanced histologic type and presence of tumor necrosis. These results reflect the increase in total mutation frequency associated with the malignancy of tumor type. High-grade SCLC and LCNEC have a higher somatic mutation rate (> 7 per million base pairs) than the lung carcinoids (0.4 per million base pairs) [15]. Thus, the high-grade carcinoma might secret stronger neoantigens to generate higher immunogenicity to recruit more cytotoxic T lymphocytes to kill non-self-components. Then, PD-L1, which is expressed by tumor cells, is increased to resist the immune protection function of CD8+ TILs.

Inflammatory cytokines, particularly interferon gamma (INF-г), can up-regulate PD-L1 expression in various cell types including tumors. In both melanoma and squamous cell carcinoma of head and neck (HNSCC), INF-г has been highlighted as a major cytokine driving PD-L1 expression [16, 17]. This indicates that PD-L1 is upregulated in tumor cells in response to secretion of INF by CD8+ T cells, as an adaptive immune-resistance mechanism that suppresses local effector T-cell function [18–20]. The results detailed above indicate that positive expression of PD-L1, especially in tumor cells, is associated with a trend toward decreased OS and PFS of PNETs. Such a trend is consistent with results previously published [21]. Moreover, we found that PD-L1 expression was predominantly present at the tumor-stroma margin, in the presence of accumulated stromal CD8+ T cells. This indicates that the PD-L1 expression manifests as a result of forces favoring tumor progression, while stromal CD8+ T cell infiltration manifests as a result of the body’s efforts at immune protection [17].

We also found that the higher CD8+ T cell density, the higher it was significantly associated with the absence of vascular invasion, negative lymph node metastasis and lower clinical staging. These findings may partially explain why stromal presence of CD8+ T cell infiltration was an independent factor for favorable OS and PFS in neuroendocrine carcinomas of the lung.

Notably, we detected PD-L1 expression in 45.3% (72/159) of neuroendocrine carcinoma of the lung. Our result was inconsistent with the study conducted by Schultheis et al. [13]. In that study, they found that tumor-infiltrating macrophages (TIMs) and tumor-infiltrating lymphocytes (TILs) rather than tumor cells showed PD-L1 protein expression. Additionally, the positive rate of PD-L1 in our study was lower than the result previously reported [21]. The inconsistent results can be attributed to the different antibodies and different criteria of scoring used in different studies.

Nonetheless, our study reports several important findings that may have implications for targeting PD-L1 immunotherapy in PNETs. First, we proved that CD8+ T cell infiltration was positively correlated with PD-L1 expression, which indicates an adaptive immune resistance-type mechanism that can also be observed in PNETS. Second, while the PD-L1 expression was observed in 16.4% of the tumor cells, 39.6% cases of PNETs showed PD-L1 expression in the immune cells at the invasive stroma-tumor margin. Though it has increasingly been recognized that PD-L1 expression on immune cells at the invasive margin represents evidence of an immune resistance mechanism, in our study, the PD-L1 expression on tumor cells was of great importance to predict the prognosis [18, 22]. Third, we showed a trend toward the correlation between expression of PD-L1 and decreased OS and PFS. This may provide a theoretical basis for anti-PD-L1 immunotherapy in PNETs. Moreover, increased CD8+ TILs density in stroma was an independent prognostic factor for improved OS and PFS. When sufficient T cells present in the tumor induce an adaptive expression of PD-L1, the PD-L1+/CD8+ patients may be most likely to respond to anti-PD-1/PD-L1 therapy. Therefore, there is a need for quantitative assessment of TIL and PD-L1 presence in resected samples to produce the desired predictive information [23].

Use of traditional treatments such as chemotherapy and radiotherapy may lead to the emergence of tumor-associated antigens, which was released from the apoptotic tumor cells [24]. If the initial traditional therapy can effectively present the tumor cells as foreign tumor antigens, it is obvious that subsequent immunotherapy is possible to be a favorable endeavor [25]. Recent studies have reported that the presence of TILs may predict a positive response to neoadjuvant chemotherapy [26]. We, therefore, sought to select patients who were more available for the immunotherapy, to maximize the benefit derived from the chemotherapy/radiotherapy-immunotherapy combined strategies.

Though we believe our study illustrates the value of PD-L1 as a potential prognostic marker, there were some limitations. First, this study involved a relatively small number of patients. This small sample size may have resulted in selection bias. Second, mature survival information was limited as the follow-up duration in our study was not long enough to fully evaluate 5-year survival rates.

## Conclusion

We found that PD-L1 was expressed in half of the PNETs and associated with necrosis, high pathologic grade and advanced histologic type. PD-L1 expression was observed primarily in immune stromal cells but showed no associations with OS or PFS. In contrast, higher levels of CD8+ TILs observed in stroma were associated with the absence of vascular invasion, negative lymph node metastasis, lower clinical staging and was demonstrated to be an independent factor for improved OS and PFS. Our results may highlight a promising method for the selection of patients with PNETs who are most likely to benefit from immunotherapy in the future.

## Abbreviations

AC: Atypical carcinoid
APCs: Antigen-presenting cells
CTLs: Cytotoxic lymphocytes
FFPE: Formalin-fixed paraffin-embedded
HNSCC: Squamous cell carcinoma of head and neck
IHC: Immunohistochemistry
INF-г: Inteferon gamma
LCNEC: Large cell neuroendocrine carcinoma
mAbs: Monoclonal antibodies
OS: Overall survival
PD-L1: Programmed death-ligand 1
PFS: Progression free survival
PNETs: Pulmonary neuroendocrine tumors
SCLC: Small cell lung carcinoma
TC: Typical carcinoid
TILs: Tumor-infiltrating lymphocytes
TIMs: Tumor-infiltrating macrophages
